# Tomato plant response to heat stress: a focus on candidate genes for yield-related traits

**DOI:** 10.3389/fpls.2023.1245661

**Published:** 2024-01-08

**Authors:** Salvatore Graci, Amalia Barone

**Affiliations:** Department of Agricultural Sciences, University of Naples Federico II, Portici, Naples, Italy

**Keywords:** *Solanum lycopersicum*, climate change, high temperatures, reproduction, heat tolerance

## Abstract

Climate change and global warming represent the main threats for many agricultural crops. Tomato is one of the most extensively grown and consumed horticultural products and can survive in a wide range of climatic conditions. However, high temperatures negatively affect both vegetative growth and reproductive processes, resulting in losses of yield and fruit quality traits. Researchers have employed different parameters to evaluate the heat stress tolerance, including evaluation of leaf- (stomatal conductance, net photosynthetic rate, Fv/Fm), flower- (inflorescence number, flower number, stigma exertion), pollen-related traits (pollen germination and viability, pollen tube growth) and fruit yield per plant. Moreover, several authors have gone even further, trying to understand the plants molecular response mechanisms to this stress. The present review focused on the tomato molecular response to heat stress during the reproductive stage, since the increase of temperatures above the optimum usually occurs late in the growing tomato season. Reproductive-related traits directly affects the final yield and are regulated by several genes such as transcriptional factors, heat shock proteins, genes related to flower, flowering, pollen and fruit set, and epigenetic mechanisms involving DNA methylation, histone modification, chromatin remodelling and non-coding RNAs. We provided a detailed list of these genes and their function under high temperature conditions in defining the final yield with the aim to summarize the recent findings and pose the attention on candidate genes that could prompt on the selection and constitution of new thermotolerant tomato plant genotypes able to face this abiotic challenge.

## 1 Introduction

**Figure 1:**
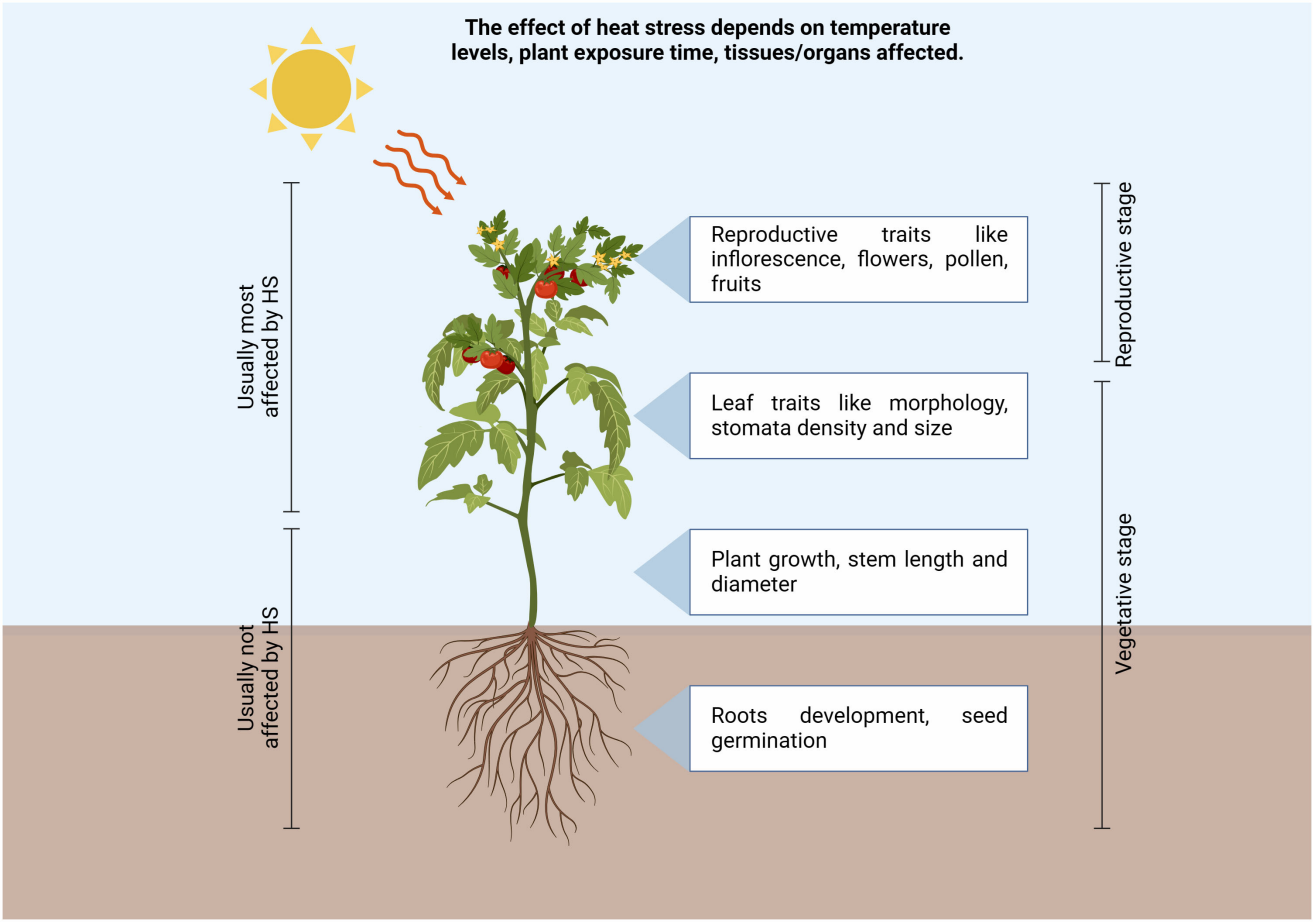
Schematic representation of high temperatures environmental conditions affecting tomato plant growth and cultivations in different tissues. Increase in temperatures above the optimum dramatically affect reproductive and vegetative stages, inducing leaf trait modifications, alteration of flower and pollen development, thus resulting in a reduction of the fruit set with consequent yield losses. (Created with BioRender.com).

Climate change caused by a rise in temperatures under natural conditions is predicted to significantly affect plant growth and development, comporting a dramatical reduction in crop productivity (Bita and Gerats, 2013). In 2017 the global average surface temperature of the earth has increased between 0.8°C and 1.2°C above the pre-industrial level, resulting in a plethora of ecological, economic and societal impacts (Masson-Delmotte et al., 2019). As a whole, it was predicted that the global agricultural productivity will decline between 3 to 16% by 2080 because of climate change (Cline, 2007). Tomato (*Solanum lycopersicum* L.) is one of the most important horticultural crops worldwide. In 2019 over 5 million hectares were allocated for tomato production, which was of around 250 million tons worldwide, and the countries with the highest production were China, India, and Turkey, which represented over 60% of world tomato production (retrieved from http://www.fao.org/faostat/en/#home, FAO–Food and Agriculture Organization of the United Nations, 2019), indicating its economic relevance for both fresh and processed consumption. Tomato plant is a sessile organism and is constantly challenged by a wide range of environmental stresses, such as drought, salt, and temperature changes, with consequent yield losses. All these stresses induce the production of reactive oxygen species (ROS), which imply oxidative stress and cell death (ul Haq et al., 2019). Plants might experience heat stress (HS) when subjected to high temperatures for a period of time higher than a threshold level, and this could permanently impair their growth and development. On the other hand, thermotolerance refers to the capability of plants to survive in extremely high or low environmental temperature conditions and produce commercial yield (Alsamir et al., 2021). Thermotolerance is generally divided into basal thermotolerance, namely the inherent ability to survive above the optimal growth temperatures, and acquired thermotolerance, which refers to the ability to cope with lethal high temperatures, following acclimatization at moderately high temperatures prior to a subsequent more severe HS; by contrast, basal thermotolerance refers to the absence of heat acclimation or pre-adaption (Larkindale et al., 2005; Suzuki et al., 2008; Stief et al., 2014; Sun et al., 2019). Since the increase of temperatures above the optimum usually occurs late in the growing tomato season, at least in the Mediterranean area, this dramatically affect reproductive stages (**Figure 1**), even though they could also impact vegetative stages, inducing leaf trait modifications. During the reproduction phases, both the time of exposure to heat stress and the temperature levels comport negative effects, resulting in flower abscission, impaired growth of stamens and pistils, poor pollen germination and altered pollen tube development with consequent low levels of fruit set and losses in the final yield (Ayenan et al., 2019; Raja et al., 2019).

**Figure 2:**
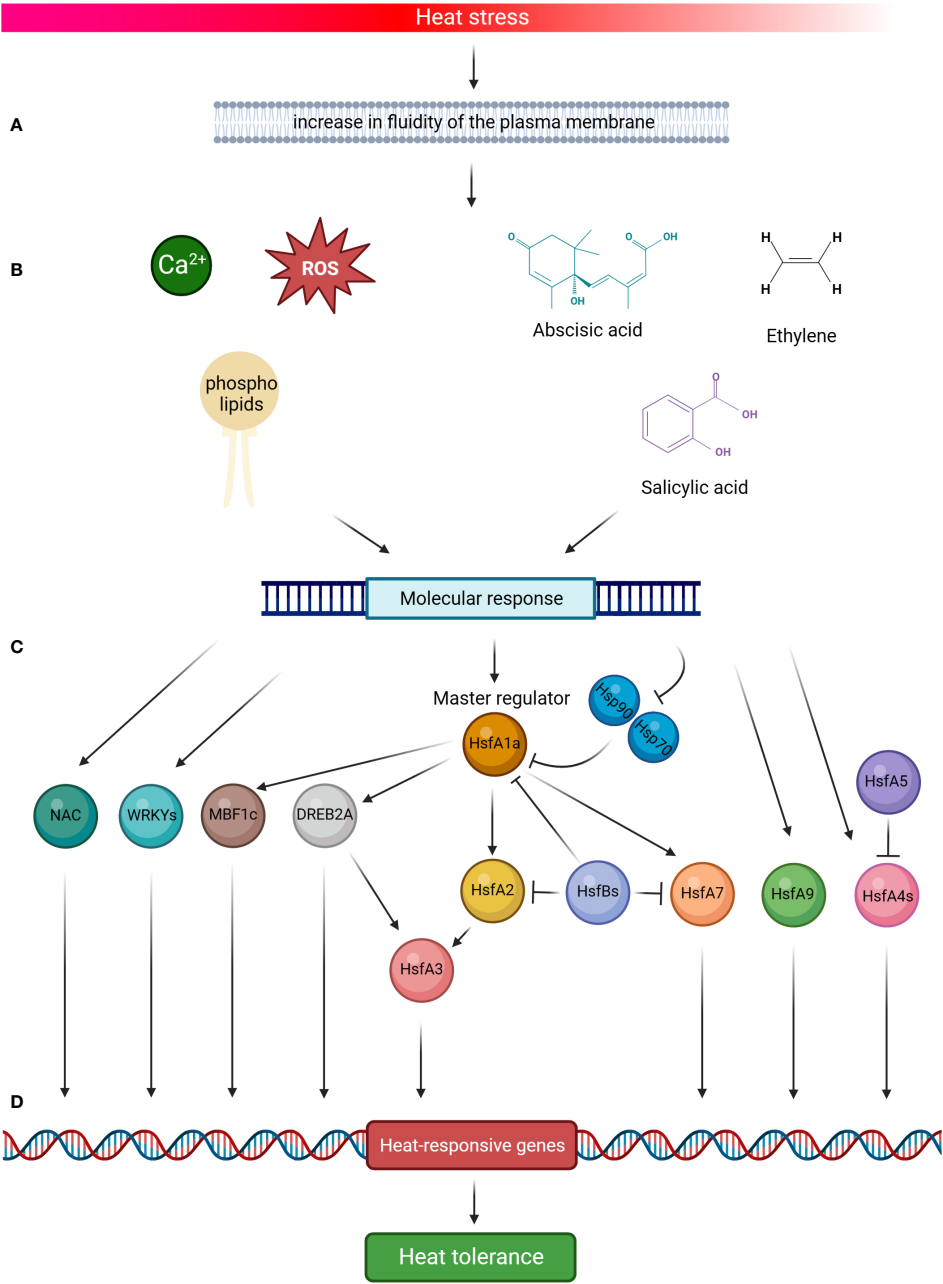
Schematic representation of tomato heat stress (HS) response. High temperature signalling pathways are activated by the increase in fluidity of the plasma membrane **(A)**. This comports the activation of the channels that mediate the entrance of calcium ions (Ca2+) into the cells, the accumulation of Reactive Oxygen Species (ROS), the remodelling of membrane phospholipids, and the role of phytohormones in determining the onset of the molecular response **(B)**. HsfA1a is the master regulator of this response and is activated by HS **(C)**, which elicits the dissociation of HsfA1s from the two heat shock proteins Hsp70 and Hsp90, thus leading its action to start. In the cascade molecular events, HsfA1 directly activates HsfA2, HsfA7, DREB2A and MBF1c, all TFs promoting the thermotolerance by the induction of HS-related genes **(D)**. Both HsfA2 and DREB2A also induce the expression of HsfA3. HsfA4s act as potent enhancers of HS gene expression, whereas HsfA5 specifically inhibits HsfA4s activity. In addition, the signalling system **(A, B)** induces the molecular response of HsfA9, NAC and WRKYs TFs, which function as activators on the promoters of several Hsps. By contrast, HsfBs are transcriptional repressors of the activities of HsfA1s, HsfA2 and HsfA7. Altogether, this complex mechanism contributes to the tomato HS response (Created with BioRender.com).

Tomato plants respond to HS by activating developmental, physiological and biochemical modifications under the expression of stress-responsive genes (Guo et al., 2016). The molecular response includes stress signal perception, signal transduction to cellular components, gene expression, and, finally, metabolic changes inducing stress tolerance (Agarwal et al., 2006). The complex signalling system, that triggers the response to high temperatures, involves the role of Reactive Oxygen Species (ROS), calcium ions (Ca^2+^) flux, phospholipids and phytohormones, and their cross talk activates different classes of transcription factors and the consequent cascade in determine the heat-responsive genes reaction (**Figure 2**). In a simplified model, the increase in fluidity of the plasma membrane due to HS comports the activation of the channels that mediate the entrance of Ca2+ into the cells, the accumulation of ROS, the remodelling of membrane phospholipids and the increase of Phosphatidyl inositol 4,5-bisphosphosphate (PIP2) and phosphatidic acid (PA), which act as key mediators of signalling pathways, the role of phytohormones like abscisic acid (ABA), salicylic acid (SA) and ethylene, which determine the onset of the molecular response through the expression of heat-responsive genes (Choudhury et al., 2017; Nievola et al., 2017; Medina et al., 2021; Haider et al., 2022; Huang et al., 2022; Pan et al., 2019a; Qu et al., 2013).

In the present review, we have focused on the tomato molecular response to HS during the reproductive stage, with an emphasis on the genes involved in this complex mechanism and their interactions. This work aimed not only to better clarify and resume the old and novel findings published on this issue but also to provide a comprehensive list of genes, among which Heat Shock Factors (Hsfs), Heat Shock Proteins (Hsps), flower-, pollen- and fruit set related, that might be involved in the tomato HS response.

## 2 Heat shock factors

**Figure 3:**
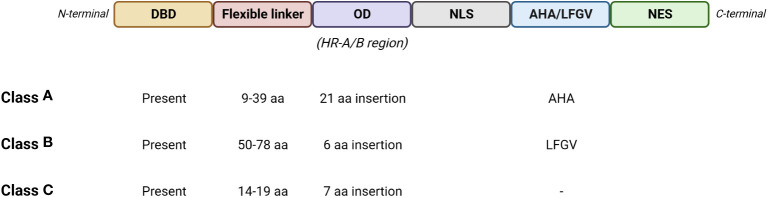
Schematic representation of the basic structure of Hsfs with the main features of the three classes. DBD, DNA binding domain; OD, oligomerization domain; NLS, nuclear localization signal domain; AHA, short activator peptide motif; LFGV, LFGV-tetraptide repressor motif; NES, nuclear export signal domain. (Created with BioRender.com).

Tomato HS response is governed by a network of Hsfs (**Figure 2**), which play a key role by detecting stress signalling and regulating the expression of several stress-responsive genes (Guo et al., 2016). The gene expression is regulated by the binding of Hsfs with heat stress elements (HSEs) distributed in the promoter regions of the targeted genes. HSEs are generally found in HS responsive genes and consist in a palindromic consensus sequence presenting a purine- and a pyrimidine-rich modules (5’-AGAAnnTTCT-3’) (Nover et al., 2001; Fragkostefanakis et al., 2015). Hsfs molecular structure presents: I) a N-terminal DNA binding domain (DBD) showing a central helix-turn-helix motif that binds HSEs in the promoter regions of the targeted genes; II) a oligomerization domain harboring a bipartite heptad pattern of hydrophobic amino acid residues (HR-A/B region); III) a flexible linker of variable length (15-80 amino acids) that connects HR-A/B to DBD; IV) a intracellular nuclear localization signal domain (NLS); V) a nuclear export signal domain (NES) and VI) a C-terminal short activator peptide motif (AHA) that confers transcriptional activator function to Hsfs (Baniwal et al., 2004; Guo et al., 2008; Sakurai and Enoki, 2010; Scharf et al., 2012; Fragkostefanakis et al., 2015). Three Hsfs classes (A, B and C) were identified, based on the number of amino acids present into the HR-A/B region and the length of the flexible linker (**Figure 3**) (Nover et al., 1996; Scharf et al., 2012). HsfAs show an insertion of 21 amino acid in the region within HR-A and HR-B and a flexible linker ranging from 9 to 39 ones, HsfBs consist in 6 amino acid residues in HR-A/B region and 50-78 in the flexible linker, while HsfCs present 7 amino acid residues in HR-A/B region and from 14 to 19 in the flexible linker (Nover et al., 1996; Nover et al., 2001; Scharf et al., 2012). In addition, HsfAs present AHA motifs in the C-terminal, formed of aromatic, large hydrophobic and acidic amino acid residues, and serving as transcriptional activator, while HsfBs comprise a characteristic LFGV-tetraptide motif, which acts as repressor domain (Fragkostefanakis et al., 2015; Guo et al., 2016). Little is known about HsfCs, which may play an active role in regulating plant heat tolerance (Zhuang et al., 2018).

Twenty-seven Hsf genes were reported in tomato (Yang et al., 2006; Scharf et al., 2012; Berz et al., 2019) among which 15 HsfAs, eight HsfBs, one HsfC and three Hsf-like (**Supplementary Table 1**). These genes absolve to different functions (**Table 1**).

**Table 1:** List of Heat Shock Factors (Hsfs) involved in the tomato molecular response to heat stress. Their functions are also reported.

Hsf	Function	Reference
HsfA1s	Master regulator	El-Shershaby et al., (2019)
HsfA2	Promote tomato acquired thermotolerance	Liu et al. (2011); Yoshida et al. (2011)
HsfA3	Response to different stresses	Bharti et al. (2004)
HsfA4s	ROS sensor	Qu et al. (2013)
	Enhancer of heat stress gene expression	Baniwal et al. (2007); von Koskull-Döring et al. (2007)
HsfA5	Repress the activity of HsfA4	Baniwal et al. (2007); von Koskull-Döring et al. (2007)
HsfA6s	Promote tomato acquired thermotolerance	Huang et al. (2016)
	Responce to ABA heat-induced genes	Huang et al. (2016)
HsfA7	Enhancer of heat stress gene expression	Mesihovic et al. (2022)
HsfA8	ROS sensor	Li et al. (2018)
HsfA9	Activator on the promoters of several Hsps	Kotak et al. (2007)
HsfB1	Co-activator of HafA1a	Fragkostefanakis et al. (2019)
	Repressor of heat stress responsive genes	Fragkostefanakis et al. (2019)
HsfC1	Involved in salinity, oxidative stress tolerance and plant thermotolerance	Haider et al. (2022)

Generally, only members of the HsfA1 subfamily are reported to act as master regulators in stress response and thermotolerance (Liu et al., 2011; Yoshida et al., 2011). In tomato, among four HsfA1, HsfA1a (*Solyc08g005170*) solely acts as master regulator. El-Shershaby et al., (2019) demonstrated that HsfA1a is constitutively expressed under control and HS conditions in all the investigated tissues while HsfA1b (*Solyc03g097120*) showed a high variation in gene expression and was strongly induced in all fruit stages. By contrast, HsfA1c (*Solyc08g076590*) and HsfA1e (*Solyc06g072750*) generally showed low expression levels except in red ripe fruits, mainly indicating their involvement in the regulation of developmental processes. HsfA1a regulates the initial transcriptional activation and nuclear retention of chaperones and additional Hsfs, among which HsfA2 (*Solyc08g062960*), that are involved in maintenance and attenuation of the HS response, thus promoting the tomato acquired thermotolerance (Liu et al., 2011; Yoshida et al., 2011). HsfA2 is strongly expressed during the early stages of anther and pollen development and is involved in the development activity and in the control of stress-regulation genes. Indeed, Fragkostefanakis et al. (2016) demonstrated that HsfA2 suppression reduced the viability and germination rate of pollen exposed to HS during the stages of meiosis and microsporogenesis but had no effect on more advanced stages, thus supporting its role in maintenance of thermotolerance. In addition, Hu et al. (2020), investigating the genotypic variation of wild and cultivated tomato in thermotolerance, showed that the progressive sensitivity to high temperatures was associated to a polymorphism within the second intron of HsfA2 sequence. In the wild species, the intron splicing promoted the early stress response reducing the short-term acclimatation and thermotolerance, thus concluding that the HsfA2 in cultivated tomato reduced its ability in a rapid HS response enhancing the short-term acclimatation ability. HsfA3 (*Solyc09g009100*) is constitutively expressed in the cytoplasm under control and in the nucleus under HS conditions (Bharti et al., 2000). It is involved in the response to different stresses, among which drought and heat. Sakuma et al. (2006) showed that it is regulated by DREB2A gene in *Arabidopsis thaliana*, a transcription factor involved in regulation of dehydration-responsive genes. Over-expression of DREB2A promoted the induction of HS related genes, including HsfA3, comporting higher tolerance to HS treatments, whereas DREB2A knockout mutants showed reduced thermotolerance (von Koskull-Döring et al., 2007). Tomato HsfA4s (*Solyc02g072000*, *Solyc03g006000* and *Solyc07g055710*) have been reported to act as potent enhancer of HS gene expression, whereas HsfA5 (*Solyc12g098520*) specifically inhibit HsfA4s activity (Baniwal et al., 2007; von Koskull-Döring et al., 2007). In addition, studies conducted on *Arabidopsis thaliana* revealed that HsfA4a (*Solyc03g006000*) acts as sensor of ROS produced under HS (Qu et al., 2013). HsfA6s (*Solyc06g053960* and *Solyc09g082670*) also improve tomato acquired thermotolerance under HS and their respond to abscisic acid (ABA) heat-induced genes. Huang et al. (2016) demonstrated that ABA treatments activate the ABA signalling master effector ABSCISIC ACID–RESPONSIVE ELEMENT BINDING PROTEIN 1 (AREB1), which promoted the HsfA6s expression in *Arabidopsis thaliana*. Mesihovic et al. (2022) evidenced that, upon mild HS, alternative splicing of HsfA7 (*Solyc09g065660*) generated a stable protein isoform that regulated the activity of HsfA1a and the abundance of HS responsive genes in tomato. Moreover, Rao et al. (2022a), through GUS-aided promoter-reporter assays and VIGS silencing and transient over-expression approach, reported that both increasing HsfA7 levels and down-regulation of HsfB4a (*Solyc04g078770*) govern the thermotolerance in a heat tolerant tomato genotype. As for the HsfA4a, also the HsfA8 (*Solyc09g059520*) was proposed to function as ROS sensor to regulate the expression of HS-induced oxidation-related genes (Li et al., 2018). HsfA9 (*Solyc07g040680*) has been demonstrated to function as activator on the promoters of several Hsps. It was exclusively expressed in late stages of seed development in *Arabidopsis thaliana* and its expression is regulated by the seed-specific transcription factor ABSCISIC ACID-INSENSITIVE 3 (ABI3) (Kotak et al., 2007). Unlike HsfAs, class B Hsfs act as repressor of HS responsive genes. Fragkostefanakis et al. (2019) showed that HsfB1 levels under control conditions were low, increased after HS thus decreasing till the basal level during the recovery process. HsfB1 (*Solyc02g090820*) over-expression under non-stress conditions generated a tomato phenotype with aberrant growth and development but with increased thermotolerance, by promoting the accumulation of HS related genes, thus highlighting its role as co-activator of HafA1a. However, its suppression under HS resulted in a higher induction of Hsps related to the activity of the other Hsfs, thus showing an enhanced plant thermotolerance and also highlighting its role as transcriptional Hsfs repressor. In contrast to class A and B Hsfs, despite less is known about HsfC (*Solyc12g007070*), it was reported to play a role in salinity, oxidative stress tolerance and plant thermotolerance (Haider et al., 2022).

## 3 Other classes of transcriptional factors

Other TFs, such MBF1, NAC, WRKY, MYB, bZIP and DREB, are known to participate in plant growth, development and stress response, and are also involved in the regulation of heat-responsive genes (Tolosa and Zhang, 2020). Among these, the MBF1c (*Solyc07g062400*) over-expression in *Arabidopsis thaliana* was reported to enhance thermotolerance (Suzuki et al., 2008; Zhang et al., 2019). Yoshida et al. (2011) reported that HsfA1 regulates HS-induced MBF1c expression. Liang et al. (2015) highlighted the positive regulatory role of the SlNAC1 (*Solyc04g009440*) to improve tomato tolerance under high temperatures. Indeed, its downexpression comported a reduced accumulation and activity of Hsps and plant antioxidant enzymes, respectively, thus resulting in the high accumulation of ROS. Among the WRKY TFs, Wang et al. (2022) identified the SlWRKY3 as positive regulator of HS response in tomato. Its over-expression led to an increased thermotolerance and decreased ROS accumulation. In addition, they demonstrated that under HS, SlWRKY3 (*Solyc02g088340*) binds the promoter region of SlGRXS1 gene cluster, which are involved in ROS scavenging, thus promoting the tomato HS response. Meng et al. (2015) posed their attention on the LeAN2 gene that encodes an R2R3-MYB TF (*Solyc10g086290*) involved in anthocyanin regulation, observing that its over-expression in transgenic tomato plants improved the plant thermotolerance through higher net photosynthetic rate, higher non-enzymatic antioxidant activity and maximal photochemical efficiency of photosystem II, and less accumulation of ROS compared to the wild type under HS. Li et al. (2015) investigated the expression level of 26 tomato bZIPs and identified that SlbZIP10 (*Solyc01g109880*), SlbZIP32 (*Solyc04g072460*) and SlbZIP33 (*Solyc04g078840*) genes were up-regulated in leaf and root tissues under HS, even if further investigations would be conducted to elucidate their role in tomato thermotolerance. Finally, it is reported that dehydration-responsive element binding (DREB) transcription factors play crucial regulatory roles in abiotic stress. Mao et al. (2020) highlighted that the SlDREBA4 (*Solyc06g066540*) regulated the downstream gene expression of many heat shock proteins (Hsp) under HS. It also induced the expression of biosynthesis genes in jasmonic acid (JA), salicylic acid (SA), and ethylene (ETH).

## 4 Heat shock proteins

The HS signal perception by Hsfs leads to an increased expression of several Hsps. These are essential in maintaining balanced cell internal conditions under optimum and stress conditions and their main functions involve protein folding, unfolding and transport, thus maintaining plant homeostasis (Khan et al., 2021). Hsps are generally grouped into five classes based on their molecular weight in kilo Dalton (kDa), such as Hsp100, Hsp90, Hsp70, Hsp60 and small Hsps (sHsps) (Wang et al., 2004; Kotak et al., 2007; ul Haq et al., 2019) (**Table 2**).

**Table 2:** List of Heat Shock Protein (Hsps) tomato classes. The number of genes involved in each Hsp class and their roles are also reported.

Hsp class	Hsp no	Role	Reference
Hsp100	6	Disaggregation and degradation of non-functional but potentially harmful proteins	Wang et al. (2004)
Hsp90	7	Protein folding	Fragkostefanakis et al. (2015); Wang et al. (2004)
		Signal transduction network	Fragkostefanakis et al. (2015); Wang et al. (2004)
		Co-regulation of HS gene expression	Hahn et al. (2011)
Hsp70	25	Maintain internal cell stability	Usman et al. (2017)
		Co-regulation of HS gene expression	Hahn et al. (2011)
Hsp60	18	Protein folding	Mahmood et al. (2010)
sHsp	157	Maintain protein functional conformation	Arce et al. (2020); Wu et al. (2022)

The Hsp100 plays an essential role in plant response to high temperatures performing disaggregation and degradation of non-functional but potentially harmful proteins (Wang et al., 2004). Yang et al. (2006) cloned the LeHsp100 (*Solyc02g088610*) gene homolog from tomato, localized in the chloroplast, highlighting its contribute to the acquisition of thermotolerance under HS. Indeed, LeHsp100 is not detected under normal conditions but is induced by HS. Unless little is known about this gene family, Gul et al. (2021) conducted a genome wide analysis thus identifying six putative Hsp100 genes (**Supplementary Table 2**), among which four were found in chloroplast (*Solyc02g088610*, *Solyc03g117950*, *Solyc03g118340* and *Solyc12g042060*), one in mitochondria (*Solyc06g011400*) and one in the cytoplasm (*Solyc03g115230*). Even these authors indicated the essential role of chloroplastic LeHsp100 in acquired thermotolerance and HS response in tomato planta. As for the Hsp90 family, it consists of at least seven genes distributed on 6 tomato chromosomes (**Supplementary Table 2**) (Zai et al., 2015). Their main function is to manage the correct protein folding. In addition, they are also involved in signal transduction network, protein degradation and trafficking (Wang et al., 2004; Fragkostefanakis et al., 2015). Even the Hsp70 family has a key role in maintaining internal cell stability. This group belong 25 tomato genes (**Supplementary Table 2**), most of which were involved in HS response while others were constitutively expressed and were reported as 10 kDa heat shock cognate (Hsc70) (Usman et al., 2017; Vu et al., 2023). Hahn et al. (2011) proposed a crosstalk activity between the cytosolic Hsp90 and Hsp70 chaperones in co-regulating HS gene expression within a network interaction with HsfA1, HsfA2 and HsfB1. Particularly, they identified two general mechanisms of interaction: I) Hsp70 repressed HsfA1 and the co-activator function of HsfB1, while Hsp90 promoted the HsfB1 binding activity; II) Hsp90 modulated the HsfA2 and HsfB1 transcript abundance and degradation. Under control conditions, HsfA1s activities were repressed through the inhibitory crosstalk activity between Hsp70/Hsp90 (Andrási et al., 2021). Exposure to HS triggers protein deformation/denaturation. Both Hsp70/Hsp90 act as molecular chaperons and bind to denatured proteins to restore protein homeostasis inside the cell (Scharf et al., 2012; Jacob et al., 2017; Andrási et al., 2021). The Hsp60 family, also called chaperonins, helps in protein folding and subunit assembly. Despite the functional characterization of plant chaperonins is limited, they are important in assisting plastid proteins like Rubisco (Mahmood et al., 2010). Eighteen genes belonging to this family were found from Fragkostefanakis et al. (2015) (**Supplementary Table 2**) as orthologues of those reported for *Arabidopsis thaliana*. Finally, sHsps protect plant cells by preventing protein degradation and maintaining their functional conformation (Arce et al., 2020; Wu et al., 2022). Unlike other Hsps, their activity is independent of ATP and binds to protein denatured by stress, preventing the irreversible denaturation and working on its refolding (Waters and Vierling, 2020). Generally, sHsps can be classified based on their molecular weight (ranging from 12 to 42 kDa), subcellular localization and homology with amino acid sequences. According with this, six classes have been identified based on their localization: mitochondria (MTI and MTII), chloroplasts (CP), cytoplasmic/nuclear (CI-CVI), endoplasmic reticulum (ER), plastids (P) and peroxisome (PX) (Waters and Vierling, 2020; Sun et al., 2021; Wu et al., 2022). Among these, the ones located in mitochondria, chloroplast and cytoplasm are reported to be mostly involved in HS response (Zhang et al., 2016). sHsps share a conserved 80-100 amino acid C-terminal domain called the α-crystallin domain (ACD). Krsticevic et al. (2016), based on the presence of a conserved alpha-crystallin domain (ACD or Hsp20 domain), reported 33 sHsp20 genes, while 42 were identified from Yu et al. (2016); in addition, Fragkostefanakis et al. (2015) identified 111 sHsp40s (**Supplementary Table 2**). Zhuang et al. (2020) identified the SlWHY1 (*Solyc05g007100*) gene, which was induced by HS and involved in plant thermotolerance. During this process, this gene induces the upregulation of SlHsp21.5A (*Solyc03g113930*), encoding an endoplasmic ER-sHsp, thereby promoting thermotolerance in tomato through decreasing ROS content and increasing soluble sugar content to protect membrane stability. In another work, Wang et al. (2020) demonstrated that the tomato Hsp40 functions as a chaperone to protect the synthesis of melatonin, a molecule involved in regulation of abiotic tolerance under HS, by the regulation of the SlSNAT (*Solyc10g074910*) gene in the chloroplast. Arce et al. (2018) further highlighted the role of sHsps in tomato thermotolerance by studying the expression and interaction of Hsps in protoplast cells, both with and without HsfA2 under two different HS conditions. Based on activation or repression of HsfA2, a critical regulator of Hsps, distinct sHsps were upregulated, evidencing their role in HS response. In addition, studies of protein–protein interactions between the sHsp family and other HS response proteins (such as Hsp70, Hsp90, and MBF1c) showed that a high number of sHsps were able to mediate the alternate stress responses via a regulatory subnetwork independent of HsfA2.

## 5 Flower and flowering

**Figure 4:**
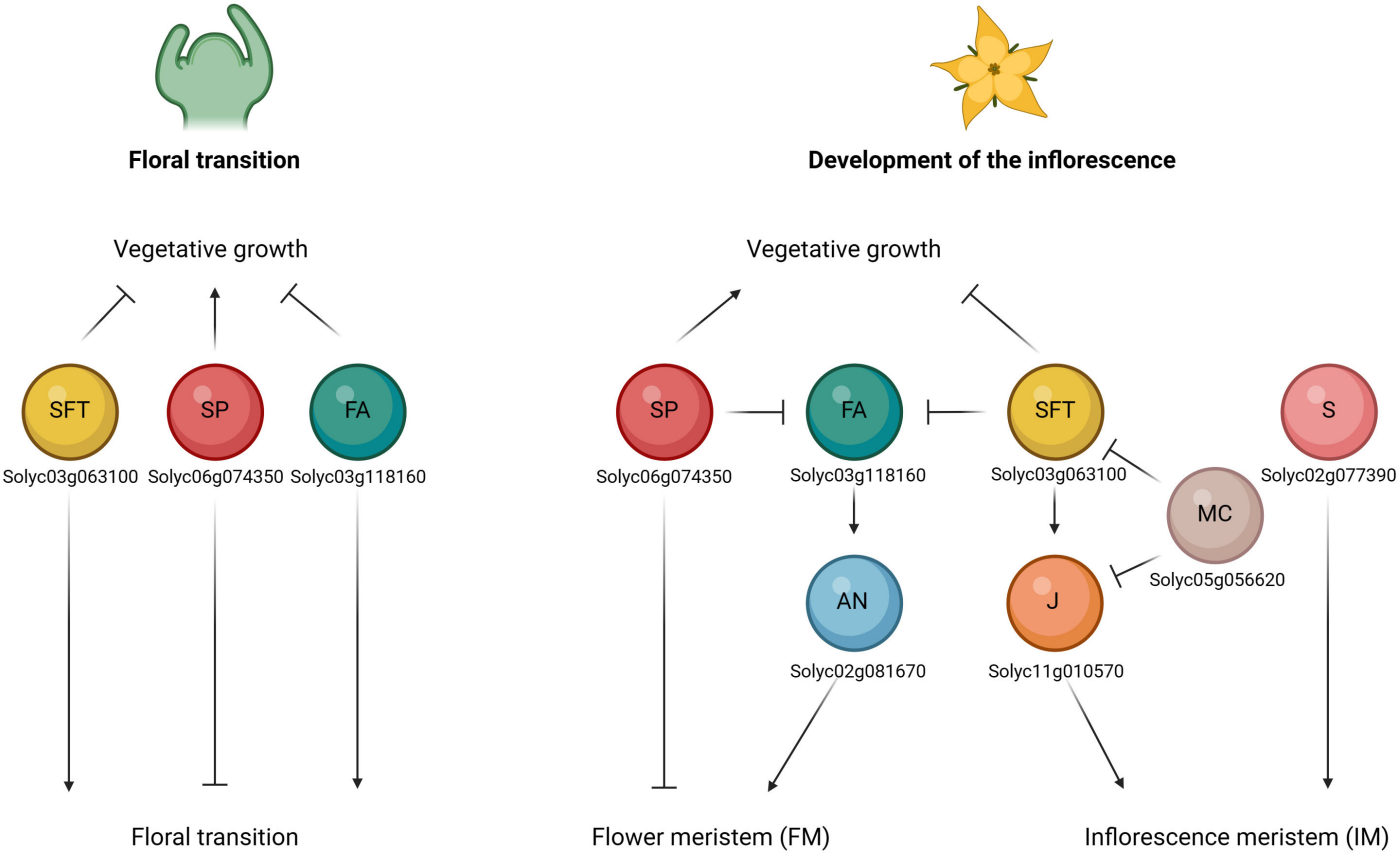
Schematic representation of the interaction of flower-related genes during the floral transition (on the left) and the development of the inflorescence stages (on the right). Floral transition of the shoot apical meristem (SAM) is promoted by upregulation of FALSIFLORA (FA) in the meristem and by systemic SINGLE FLOWER TRUSS (SFT) signal, which both repress vegetative growth. SELF PRUNING (SP) plays an antagonistic role and regulates the vegetative growth. Development of the inflorescence involves the maturation to flower meristem (FM) and inflorescence meristems (IM) fates. FA and ANANTHA (AN) genes are required for the transition from SAM to FM, while SP represses it by promoting the vegetative growth. SFT represses vegetative growth in the lateral IM. JOINTLESS (J) acts synergistically with SFT and regulates inflorescence structure to prevent premature maturation of IM toward FM. By contrast, MACROCALYX (MC) represses the two genes. Lastly, also COMPOUND INFLORESCENCE (S) gene was required for the maintenance of IM activity. (Created with BioRender.com).

Tomato inflorescence architecture represents an important trait affecting the final number of flowers and fruits, thus influencing the yield production (Zheng and Kawabata, 2017). Two types of architectures can be described, based on the growth habits of the inflorescence meristem (IM), such as monopodial and sympodial (Teo et al., 2014; Zhu and Wagner, 2020). The first is characterized by the indeterminate development of the IMs which continuously generates lateral branches or flowers; while in the second case IMs terminate in flowers through the transition to floral meristems (FMs) and growth continues from a variable number of new axillary (sympodial) IMs, which repeat this process in an iterative way to form compound inflorescence shoots (Pnueli et al., 1998; Park et al., 2012). Many important genes involved in the regulation of tomato inflorescence development and flowering time have been reported, such as SINGLE FLOWER TRUSS (SFT), SELF PRUNING (SP), FALSIFLORA (FA), ANANTHA (AN), COMPOUND INFLORESCENCE (S), JOINTLESS (J), MACROCALYX (MC), JOINTLESS-2 (J-2), FRUITFULL1 (FUL1), FRUITFULL2 (FUL2), MADS-BOX PROTEIN 20 (MBP20) (Samach and Lotan, 2007). These genes are implicated in a complex network that determines the floral transition and the development of the inflorescence (**Figure 4**).

The SFT (*Solyc03g063100*) gene encodes the ortholog of *Arabidopsis thaliana* FLOWERING LOCUS T (FT) and is reported to be the main tomato gene in promoting the florigen activity. The sft mutant may alter normal tomato sympodial development and determines the transition of the inflorescence towards vegetative functioning after the development of one or few flowers. In addition, SFT is expressed in expanded leaves and its overexpression leads to early flowering in tomato (Lifschitz et al., 2006; Yuste-Lisbona et al., 2016). Conversely, SP (*Solyc06g074350*), tomato ortholog of *Arabidopsis thaliana* TEMINAL FLOWER 1 (TFL1), plays an antagonistic role by repressing the floral transition and promoting the vegetative growth and comporting a determinate habitus (monochasial cyme). Loss of function of SP gene leads to the shortening of successive sympodial segments up to the ultimate cessation of the iterative process (Thouet et al., 2008; Périlleux et al., 2019). It is expressed in young leaves and shoot apex. The balance between the SFT florigen- (floral inducer) and SP antiflorigen (inhibitor) genes regulates flowering time and the determinate or indeterminate shoot architecture (Higuchi, 2018; Jin et al., 2021). Moreover, these genes both belong to the phosphatidylethanol- amine-binding protein (PEBP) family protein, and Cao et al. (2016) identified 13 PEBP genes in the whole tomato genome, among which six were FT-like genes. Investigating their functional role, the authors found that only the SFT gene was a floral inducer, while the *Solyc05g053850*, *Solyc11g008640* and *Solyc11g008650* proteins were floral inhibitors. The two other genes found (*Solyc05g055660* and *Solyc11g008660*) were not expressed in all the investigated tomato plants tissues (leaf, cotyledon, apex, stem, flower, and root). Song et al. (2020) demonstrated that the *Solyc11g008650* FT-like gene regulated short day flowering in tomato and activated the transcription of the florigen SFT, highlighting its role in promoting the earliest flowering in the *S. pimpinellifolium* accession in comparison with the cultivated tomato, which presented a sequence deletion that led to a very short translated protein. The FA (*Solyc03g118160*) gene, homolog of *Arabidopsis thaliana* LEAFY (LFY), controls flowering time and floral meristem identity. fa mutants resulted in the conversion of flowers in secondary buds and produced highly branched inflorescence (Molinero‐Rosales et al., 1999; Zheng and Kawabata, 2017). In addition, they are not able to develop complete flowers and produce a late flowering phenotype, with an increase in the number of leaves below the first and successive inflorescences (Yang et al., 2021). SFT and FA act in parallel pathways to promote the floral transition of the shoot apical meristem and thus repressing the vegetative growth in tomato. During the inflorescence development, FA gene is required for promoting the transition of the shoot apical meristem (SAM) to floral meristem (FM), together with the AN (*Solyc02g081670*) gene. These genes are both mainly expressed in the flower meristem (Yang et al., 2021). The AN gene encodes the F-box protein ortholog of *Arabidopsis thaliana* UNUSUAL FORMATION OF ORGANS (UFO) and is reported that the loss of function of the AN gene delays flower formation, leading to additional branching and to a cauliflower-type of the meristems (Zheng and Kawabata, 2017; Yang et al., 2022). Therefore, AN and FA formed a complex to specify flower formation, while another gene named S (*Solyc02g077390*) was required for the maintenance of IM activity (Zheng and Kawabata, 2017). S encodes the *Arabidopsis thaliana* Wuschel-related HOMEOBOX 9 (WOX9) ortholog. In tomato, mutations in this gene is reported to delay the IM transition to FM, leading to branched inflorescences (Shannon and; Meeks-Wagner, 1991; Quinet et al., 2006; Zheng and Kawabata, 2017). Another gene named J (*Solyc11g010570*) is expressed in the inflorescence meristems and regulates inflorescence structure to prevent premature maturation of IM toward FM, acting synergistically with SFT (Szymkowiak and Irish, 1999; Thouet et al., 2012; Yang et al., 2022). Indeed, J is a MADS-box gene that controls inflorescence traits in tomato like the flower abscission zone by interacting with other two MADS-box transcriptional factors such as MC (*Solyc05g056620*) and J-2 (*Solyc12g038510*), the last of which was previously reported as SlMBP21 (Liu et al., 2014; Roldan et al., 2017). The j mutant showed the typical truss converted into an inflorescence made of leaves and flowers due to the resumption of vegetative meristems in place of inflorescence meristems (Szymkowiak and Irish, 1999; Thouet et al., 2012). Yuste-Lisbona et al. (2016) highlighted the interaction of MC with J and SFT in controlling floral transition and inflorescence fate in tomato: J and SFT are involved in a positive feedback loop while MC expression represses the two genes. Lastly, Jiang et al. (2022) pose their attention on four FRUITFULL-like genes such as FUL1 (*Solyc06g069430*), FUL2 (*Solyc03g114830*) and MBP20 (*Solyc02g089210*). The authors particularly highlighted the role of FUL2 and MBP20 in promoting the vegetative-to-reproductive transition and in inducing the FM maturation thus repressing the inflorescence branching, while the FUL1 is also involved in the process but its upregulation in the inflorescence and floral meristems depends on the two genes. In addition, these three genes act downstream of the key regulator such as SFT, FA and AN during the transition to reproductive phase and the establishment of inflorescence architecture.

Not only the inflorescence architecture and flowers number affect the final yield, but also the flower development and morphology. High temperatures negatively affect these traits, and one of the main problems described was the impaired growth of stamens and pistils which determines the sterility of plants. HS conditions can strongly influence the position of stigma relative to anthers, thus comporting the so‐called stigma exertion, which hampers pollination and causes fruit set failure (Pan et al., 2019a; Alsamir et al., 2021; Riccini et al., 2021). This phenotype depends on the genotype and Saeed et al. (2007) found that the length of the style of different tomato genotypes increased by 25–55% under high temperatures. Bernacchi and Tanksley (1997) investigated an interspecific mapping population derived from *S. lycopersicum* and *S. habrochaites* and identified a first major QTL on chromosome 2 that they called se2.1. This is a complex locus presenting at least five closely linked genes, among which the style2.1 controlling style length. Chen et al. (2007) reported that this gene encodes a transcription factor presenting a conserved helix-loop-helix (HLH) motif that modules the cell elongation during the development of the pistil. Indeed, its downregulation was associated with short style phenotype. Few other QTLs for stigma position have been later identified. Georgiady et al. (2002) found a QTL on chromosome 8 (sty8.1), while Gorguet et al. (2008) identified the se5.1 QTL mapping in the long arm of chromosome 5. More recently, Xu et al. (2017) identified two new QTLs on chromosomes 1 (qSP1) and 3 (qSP3) and confirmed the previously mapped se2.1. A detailed list of the QTLs recently identified by Gonzalo et al. (2020); Xu et al. (2017), Bineau et al. (2021) and Zhang et al. (2018b) and controlling stigma exertion, flower number, inflorescence architecture, anther and style length is reported in **Supplementary Table 3**. Pan et al. (2019b) investigated the stigma exertion phenomenon in the tomato cultivar Micro-Tom and they demonstrated that it is related more to shortened stamen than pistil elongation. Indeed, the different response of pectin and sugar in both stamen and pistil under HS altered the transcript abundance of cyclins and expansins and the extensibility and porosity of the cell wall, comporting different cell numbers and sizes in the two flower organs and thus their different elongation. In addition, they found that the cell division and expansion in both the organs is regulated by auxin and jasmonate (JA). Particularly, exogenous JA can effectively rescue tomato stigma exertion through regulating the JA/COI1 signalling pathway. Finally, Cheng et al. (2021) evaluated the content of five hormones with the aim of explaining their relationships with the stigma exertion. They found that the increase of IAA content promotes style growth, while ABA accumulation is negatively correlated with IAA and indirectly affects the styles length by inhibiting the content of IAA. In addition, they identified the SlLst (*Solyc12g027610*) as the key candidate gene. It encodes an ethylene receptor protein that may play a role in the heat-perception pathway during the process of regulating stigma exertion. Overexpression of SlLst can inhibit the elongation of the style.

## 6 Pollen growth and development

The final yield is influenced not only from the total number of flowers but also from the total number of fruits, whose development depends on several factors, like pollen germination and viability and pollen tube development (Alsamir et al., 2021). The main function of pollen is to transfer the male gamete into embryo sac and its viability is influenced by biotic and abiotic stresses. Among these, high temperature decreased the pollen viability, retention of pollen in the anthers and pollen germination (Razzaq et al., 2019). Recently, only a few QTLs were reported (Xu et al., 2017) (**Supplementary Table 3**). Despite less is known on genetic mechanisms of pollen development and availability under HS, several authors posed their attention on genes involved in pollen-related traits, like pollen germination, pollen tube growth and pollen fertility (**Table 3**).

**Table 3:** List of pollen related genes involved in traits such as pollen germination, viability and tube growth. The gene families, gene names and IDs and their functions are reported.

Gene family	Gene name	Gene ID	Function	Reference
CRKs	SlCRK1 - SlCRK35	*-*	Perception of external and internal stimuli and transmission of input signals to activate target genes	Liu et al. (2021)
GDSLs	SlGELP1 - SlGELP81	*-*	Pollen fertility	Sun et al. (2022)
NCED	SlNCED1	*Solyc03g121880*	Regulates endogenous ABA and gene transcript levels in the anthers	Wang et al. (2021)
LePRKs	LePRK1	*Solyc05g047570*	Influence the pollen tube growth	Gui et al. (2014)
	LePRK2	*Solyc07g017230*		
	LePRK3	*Solyc05g025780*		
	LePRK4	*Solyc12g009190*		
	LePRK5	*Solyc03g124050*		
RALFs	SlPRALF	*Solyc07g063030*	Negative regulator of pollen tube elongation.	Covey et al. (2010)
Ethylene-responsive	ER21	*Solyc04g011440*	Pollen germination, viability and sensitivity to HS	Firon et al. (2012); Jegadeesan et al. (2018)
	ER24	*Solyc01g104740*		
	MBF1	*Solyc01g104740*		

Liu et al. (2021) investigated the role of the cysteine-rich receptor-like protein kinases (CRK) gene family in tomato under abiotic stress conditions, especially HS. CRKs belong to receptor-like protein kinases (RLKs) gene family, which is involved in the perception of a variety of external and internal stimuli and to transmit the input signal to enhance the activated expression of specific target genes. The authors performed a genome-wide analysis on tomato, thus identifying 35 putative SlCRK genes. Through a transcriptome analyses of tomato fruits collected from plants after high temperature treatment at 0 h, 24 h, 48 h and 96 h, they observed SlCRK genes were mainly downregulated upon heat. Wang et al. (2021) analysed the role of ABA in the development of tomato pollen. They investigated the *Solyc03g121880* gene, also known as SlNCED1, which encodes the 9-cis-epoxycarotenoid dioxygenase (NCED), a key gene in the ABA biosynthesis. Indeed, this hormone has an important role in the development of tomato pollen. Suppression of this gene led to a downregulation of endogenous ABA and gene transcript levels in the transgenic anthers, which also comported the downregulation and upregulation in the transcription of specific genes positively and negatively related to the anther development in tomato, respectively. They demonstrated that ABA affects pollen maturation by regulating the expression of anther-specific genes. Wang et al. (2018) described in *Arabidopsis thaliana* the role of the pollen-specific leucine-rich repeat extension genes, a family of pollen tube cell wall proteins, focusing on their involvement during pollen tube growth, in maintaining pollen tube cell wall integrity and thus playing a critical role in pollen germination and pollen tube growth. Gui et al. (2014) studied the role of the tomato pollen receptor kinase LePRK1 (*Solyc05g047570*) and other members of its clade, among which LePRK2 (*Solyc07g017230*), LePRK3 (*Solyc05g025780*), LePRK4 (*Solyc12g009190*) and LePRK5 (*Solyc03g124050*). They showed that overexpression of LePRK1 influenced the pollen tube growth from tubular to blebbing thus causing drastic morphological changes in growing pollen tubes. Overexpression of LePRK2 caused pollen tube tip swelling and sometimes hockey stick–like tubes and the overexpression of LePRK3, LePRK4, or LePRK5 caused only slight swelling of the tip. Huang et al. (2014) posed their attention on the tomato stigma-specific protein 1 STIG1 gene (*Solyc03g120960*), a small cysteine-rich protein from the pistil. They conducted *in vivo* studies and they demonstrated that the STIG1 acts as a peptide signalling molecule for LePRK2 in promoting pollen tube growth by affecting cellular reactive oxygen species (ROS) production. Covey et al. (2010) identified a pollen-specific tomato rapid alkalinization factor SlPRALF (*Solyc07g063030*) gene. This gene was found to not affect pollen viability, hydration, or early germination events but acts as a negative regulator of pollen tube elongation. Another family, the GDSL esterase/lipase class, contains many functional genes playing a key role in the regulation of plant growth, response to stress and the morphogenesis of tissues and organs. In addition, these genes can respond to biotic and abiotic stresses (Sun et al., 2022). GDSLs are also involved in pollen fertility in *A. thaliana*. A knockout of GELP77 in this species caused male sterility and failure of pollen separation (Tsugama et al., 2020). Sun et al. (2022) identified through a bioinformatic approach 80 GDSL esterase/lipase family genes in tomato, coded from SlGELP1 to SlGELP81. Finally, it was demonstrated that ethylene, a gaseous plant hormone, plays a key role in tomato pollen thermotolerance. Interfering with the ethylene signaling pathway or reducing ethylene levels increased tomato pollen sensitivity to HS, whereas increasing ethylene levels prior to HS exposure increased pollen germination and viability (Firon et al., 2012). In tomato pollen, Jegadeesan et al. (2018) reported a high upregulation under HS conditions of two genes, known to be ethylene-responsive in tomato fruit: ER21 (*Solyc04g011440*), an ethylene-responsive heat shock protein 70, which showed more than 15-fold expression levels in both anthers and pollen grains, and ER24 (*Solyc01g104740*), an ethylene-responsive transcriptional coactivator MBF1 (*Solyc01g104740*), which exhibited 150-fold elevated expression levels in earlier stage of pollen maturation.

## 7 Fruit set

Fruit set is a crucial stage of development in which the ovary is transformed into fruit. In this process, plant hormones and hormone-related genes play important roles (**Table 4**).

**Table 4:** List of fruit set related genes involved in the transition of tomato ovary to fruit. The hormone classes, gene names and IDs, their functions are reported.

Hormone class		Gene name	Gene ID	Function	Reference
*Auxin*				*Acts as positive regulatory signals in early fruit development. After fertilization, an auxin signal promotes GA synthesis in the ovule*	Dorcey et al. (2009)
		SlARF7	*Solyc07g042260*	Regulates the auxin accumulation during tomato fruit growth and negative regulates fruit set until pollination and fertilization occurred	De Jong et al. (2009)
		SlIAA9	*Solyc04g076850*	Acts as negative regulator of the transition from flower to fruit	Wang et al. (2005)
		SlPIN4	*Solyc05g008060*	Acts altering the local distribution of auxin in the early stages of flower bud development, thus affecting the fruit set	Mounet et al. (2012)
		PAD1	*Solyc01g111450*	Prevents overaccumulation of IAA in unpollinated ovary thus resulting in fruit set	Matsuo et al. (2020)
*GA*				*Acts as positive regulatory signals in early fruit development. GA is transported to the pericarp to promote fruit set*	Dorcey et al. (2009)
		SlDELLA	*Solyc11g011260*	Negative regulator of GA signaling pathway	Shinozaki et al. (2018); Sun and Gubler (2004)
		SlGA20ox1	*Solyc03g006880*	Promote accumulation of GA in the ovary upon pollination	Serrani et al. (2008)
		SlGA20ox2	*Solyc06g035530*	Promote accumulation of GA in the ovary upon pollination	Serrani et al. (2008)
		SlGA20ox3	*Solyc11g072310*	Promote accumulation of GA in the ovary upon pollination	Serrani et al. (2008)
*ABA*				*Induces leaf stomata closure, triggers the activation of several stress-responsive genes and regulates the differentiation of floral organs and fruit ripening. Following fertilization, it is repressed from auxin*	Galpaz et al. (2008) *;* Lata and Prasad (2011); Zhang et al. (2009)
		SlNCED1	*Solyc03g121880*	Overexpression of SlNCED1 increases ABA level in the ovary and reduces fruit-set rate	Kai et al. (2019)
*Ethylene*				*Controls floral organ senescence, abscission layer development and fruit ripening. Following fertilization, it is repressed from auxin*	Kumar et al. (2013) *;* Shinozaki et al. (2015)
*SA*				*Plays a key role in systemic acquired resistance and hypersensitive response to HS, and contributes to basal and acquired thermotolerance. It regulates physiological processes in plants such as growth, photosynthesis, and other metabolic processes.*	Alsamir et al. (2021) *;* Dat et al. (2000) *;* Mohamed et al. (2020)

Among these, auxins and gibberellins were reported to be involved in ovary development during fruit set (Pesaresi et al., 2014; Azzi et al., 2015). The fertilization phase can generate an auxin signal in plants to promote gibberellin (GA) synthesis in the ovule, which is then subsequently transported to the pericarp to promote fruit set (Dorcey et al., 2009). Auxin and GA signaling pathways stimulate and directly activate tomato fruit sets and are the major hormones that promote fruit initiation. Indeed, they rapidly accumulate in tomato ovaries after pollination, and act as positive regulatory signals in early fruit development. In this context, SlDELLA (*Solyc11g011260*) and the SlARF7 (*Solyc07g042260*)/SlIAA9 (*Solyc04g076850*) complex mediates crosstalk between GA and auxin pathways to regulate fruit initiation (Hu et al., 2018). The GA signaling pathway is activated by the degradation of a negative regulator known as SlDELLA, through the ubiquitin 26S proteasome pathway, thus triggering GA responses (Sun and Gubler, 2004; Shinozaki et al., 2018). Moreover, the accumulation of GA in the ovary upon pollination is associated with the upregulation of SlGA20ox1 (*Solyc03g006880*), SlGA20ox2 (*Solyc06g035530*) and SlGA20ox3 (*Solyc11g072310*) genes, which encode the GA 20-oxidase biosynthetic enzymes (Serrani et al., 2008). SlARF7 is suggested to acts as a negative regulator of fruit set until pollination and fertilization, and then positively regulates the auxin accumulation during tomato fruit growth (De Jong et al., 2009). As for the SlARF7, also the SlIAA9 gene was found to act as negative regulator of the transition from flower to fruit (Wang et al., 2005)., Another gene, the PIN-formed 4 (SlPIN4), is also involved as auxin efflux carrier in fruit set. Mounet et al. (2012) evidenced that it was highly expressed in the ovary, ranging the highest value in flowering during the anthesis and then decreasing during the development of the fruit. They found that it acts altering the local distribution of auxin in the early stages of flower bud development, thus affecting the fruit set. Matsuo et al. (2020) shed lights on the role of the Pad-1 gene in unpollinated ovary, which prevent overaccumulation of IAA thus resulting in precocious fruit-set. In addition, they showed that its suppression induced parthenocarpic fruit development in tomato plants. The phytohormone abscissic acid (ABA) plays a crucial role in HS response, inducing leaf stomata closure to reduce water loss through transpiration and decreases the photosynthetic rate in order to improve the water-use efficiency, and triggering the activation of several stress-responsive genes (Lata and Prasad, 2011). In addition, it regulates the differentiation of floral organs and fruit ripening (Galpaz et al., 2008; Zhang et al., 2009). Kai et al. (2019) investigated the role of the SlNCED1 gene, a key ABA biosynthesis enzyme, through overexpression and transcriptome analysis in the tomato pistil. They found that the overexpression of this gene caused an increase in ABA concentration in the pistils thus comporting phenotypical alterations in ovary morphology and styles. In addition, the expression of most genes related to carbohydrate and lipid metabolism was significantly different during the ovary development, suggesting that carbohydrates and lipids are essential in this process. They concluded that ABA was observed to have a negative effect on fruit set. Indeed, overexpression of SlNCED1 increases ABA level in the ovary and reduces fruit-set rate. In addition, the gaseous hormone ethylene also influences the fruit set. Ethylene controls numerous aspects of plant development, including floral organ senescence, abscission layer development and fruit ripening. Following fertilization, it has been shown that ethylene is negatively regulated from auxin (Shinozaki et al., 2015). Indeed, although elements of the ethylene signaling pathway, such as ethylene response factors (ERFs) increase upon fertilization, ethylene- and ABA-related genes are repressed in concert with fruit set (Kumar et al., 2013). Salicylic acid (SA) (2-hydroxybenzoic acid) plays a key role in systemic acquired resistance and hypersensitive response to HS, and contributes to basal and acquired thermotolerance (Dat et al., 2000; Alsamir et al., 2021). It participates in the regulation of physiological processes in plants such as growth, photosynthesis, and other metabolic processes. Indeed, it is reported to increase the efficiency of photosynthesis through the higher accumulation of proline and is also known that SA stabilizes the trimers of heat shock transcription factors and contributes in their binding to the heat shock element in the promoter of HSP genes (Mohamed et al., 2020).

In addition to hormone-related genes, authors also reported the involvement of Hsfs and Hsps in determining the fruit set. Among these, Pham et al. (2020) isolated the HT7 plant mutant showing improved fruit-setting under long-term HS by testing a population of over 4000 Micro-Tom tomato mutant lines collection. The selected plant showed a higher fruit number, higher number of seeds into the fruits and total pollen grain number and viability under HS conditions than those of the wild type under both control and HS conditions. Expression analysis revealed that, after long-term exposure to HS, HT7 showed higher levels of SIHsfA1b3 and Hsp101 than the wild type, evidencing their role in HS response. Finally, Gonzalo et al. (2020) andBineau et al. (2021) identified 18 QTLs related to fruit number and fruit set. (**Supplementary Table 3**).

Another class of genes known as invertase play a major role in response to biotic and abiotic stresses and plant development and is reported to have important regulatory functions in both carbon metabolism and fruit set and development (Jin et al., 2009; Ru et al., 2017). They are mainly involved in the degradation of sucrose, which is transported from source to sink plant tissues through the phloem, to yield glucose and fructose for their utilization in sink organs. Based on their subcellular localization, various authors classified these genes in cell wall invertase (CWI), vacuolar invertase (VI) and cytosolic invertase (CI) or neutral invertase (NI) (Tymowska-Lalanne and Kreis, 1998; Sturm, 1999). In addition, these can also be classified in acid-INV (involving CWI and VI), which present an optimum pH ranging from 4.5 to 5, and alkaline/neutral INVs (A/N-INVs), which are mainly located in the cytosol and have an optimal pH in the range of 6.5-8 (Tymowska-Lalanne and Kreis, 1998; Sturm, 1999). In tomato 20 genes encoding INV were reported, among which 12 encoding acid-INVs and eight A/N_INVs (Qin et al., 2016; Coluccio Leskow et al., 2021). Shen et al. (2019) found that the transcript level of LIN5 (*Solyc09g010080*, a major CWI gene) and its invertase activity were significantly increased in style after pollination, demonstrating how styles respond to pollination for activation of CWI and sugar transporters to fuel pollen tube elongation. Liu et al. (2016) used a transgenic tomato line silenced for the CWI inhibitor gene and they found that the increase of CWI activity enhanced fruit set and suppressed the long-term moderate HS-induced programmed cell death in fruits. In addition, they reported a higher expression of Hsp90 and Hsp100 in ovaries and Hsp17.6 in fruits under HS conditions, with an auxin response consisting in a lower expression of a negative auxin responsive factor IAA9 and a higher transcript level of the auxin biosynthesis gene ToFZY6 (*Solyc09g074430*) in fruits. Coluccio Leskow et al. (2021) identified the tomato cytosolic A/N-INV NI6 (*Solyc04g081440*) whose transcript is present in leaves, stems, flowers and fruits, with high expression in sink tissues like roots and fruits. When investigating one NI6 knock-down transgenic plant, they observed that it showed impaired vegetative growth, delayed flowering and a dramatic reduction in the fruit set. The latter phenotype was determined from the high number of flower abortion.

**Figure 5:**
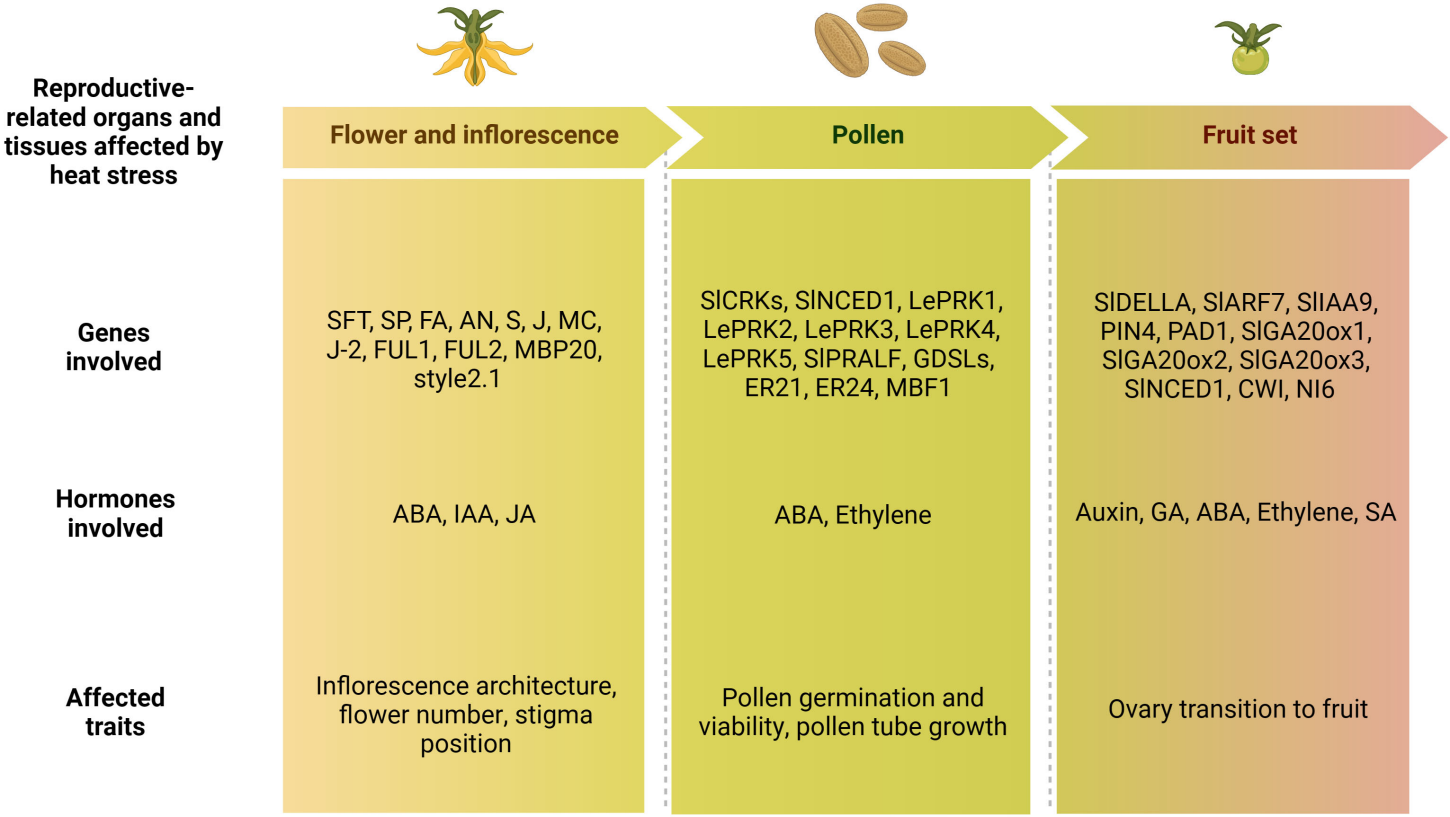
Schematic representation of the lists of flower-, pollen- and fruit set-related genes and hormones involved in the tomato heat stress response, and phenotypic traits affected by high temperatures. ABA, abscisic acid; IAA, indole acetic acid; JA, jasmonic acid; GA, gibberellin acid; SA, salicylic acid (Created with BioRender.com).

Altogether, different processes, such as flower induction, inflorescence formation, pollen development, viability and germination, as well as ovary development, style protrusion and fruit set, contribute to determine the number of fruits produced, and therefore the final yield. A synthetic list of the genes and hormones involved in these processes and previously described is reported in **Figure 5**.

## 8 Epigenetic, post-transcriptional and post-translational regulation

In the last 20 years, the effects of epigenetic modifications on plant response to external stimuli have been widely reported (Eriksson et al., 2020). In particular, it has been stated that epigenetic mechanisms are also major players of the HS thus regulating the mechanism of plant stress survival (Liu et al., 2015). Generally, epigenetics refers to the changes in gene expression that occur without DNA sequence variations (Kinoshita and Seki, 2014; McCormick, 2018). The epigenetic regulatory system includes DNA methylation, histone modification, chromatin remodelling and non-coding RNAs (ncRNAs) (Ueda and Seki, 2020; Zhao et al., 2020). DNA methylation is a chemical modification determined by the addition of a methyl group to the nitrogenous base in the DNA strand in a sequence specific manner and is performed by the DNA methyltransferases (DNMTs). The nitrogenous bases are mostly cytosines, but they also can be adenines. In addition, DNA methylation is classified as symmetrical when it occurs at CG and CHG positions, and asymmetrical when it happens at CHH position (H could be any nucleotide base other than G) (Cokus et al., 2008; Law and Jacobsen, 2010; Zhang H. et al., 2018). DNA methylation is important in plants for many biological processes since it allows to control gene expression and maintain genome integrity by silencing transposable elements (TEs) (Ikeda & Nishimura, 2015; Zhang Y.-Y. et al., 2018). Singh et al. (2021) investigated the role of DNA methylation in response to HS in the tomato mutant Slddm1b. The DDM1 (a SWI/SNF chromatin remodelling protein family member) allows DNA methyltransferases to access heterochromatin thereby facilitating DNA methylation and it was demonstrated its role in plant response to environmental conditions (Zemach et al., 2013; Sow et al., 2021). The authors showed that the DNA methylation-deficient mutant presented a better response to HS compared with the M82 control line, highlighting higher fruit set and seed set rates, and evidencing differences in the expression of HS-related genes. In response to environmental stresses, also histone proteins are subjected to several modifications like acetylation, methylation, phosphorylation, ubiquitination and biotinylation. Generally, DNA wraps around histones to forms a highly compact structure known as nucleosome. Histone alterations can modify amino acids present in the N terminal tails (like lysine and arginine), interfering in the interaction between histone and DNA and changing the packaging structure, which either activates the DNA for the transcription or makes the structure even condensed so that transcription machinery is unable to bind to it (Ohama et al., 2017; Saraswat et al., 2017; Shanker et al., 2020; Kumar et al., 2021). Aiese Cigliano et al. (2013) conducted an in silico genome analysis in tomato and identified 32 histone acetyltransferases (HATs), 15 histone deacetylases (HDACs), 52 histone methytransferases (HMTs) and 26 histone demethylases (HDMs). HATs are considered gene activators, whereas HDACs led to transcriptional repression of associated genes (Tahir and Tian, 2021). In tomato it is reported that HsfB1 recruits histone acetyltransferase 1 (HAC1) to chromatin, suggesting that the interaction of HsfB1 with HAC1 regulates gene expression and provides HS tolerance (Bharti et al., 2004). In plants, changes in chromatin architecture in response to stresses could coordinate global transcriptome modifications for appropriate cellular and physiological responses (Sun et al., 2020; Bhadouriya et al., 2021). Huang et al. (2023) demonstrated that in tomato HS induced chromatin remodeling, leading changes in the interactions between promoters and the distal regulatory elements. In addition, investigating the role of the HS master regulator HsfA1a, they found that it plays a key role in the dynamic formation of promoter-enhancer contacts and in controlling the transcriptional response at the onset of HS. Recently, more emerging ncRNAs have been found to play important roles in HS response (Ding et al., 2020; Li et al., 2023). This RNA class does not encode a protein and involves microRNAs (miRNAs), small interfering RNAs (siRNAs), long non-coding RNAs (lncRNAs), and circular RNAs (circRNAs). miRNAs present 20-24 nucleotides and are reported to negatively regulate gene expression by either mRNA degradation or translation inhibition (Rogers and Chen, 2013; Bhogireddy et al., 2021). siRNAs are either exogenous or endogenous RNAs derived from the Dicer-like (DCL) family that catalyzes the processing of double-stranded RNA (dsRNA) precursors and show approximately 21-24 nucleotides (Axtell, 2013; Bhogireddy et al., 2021). lncRNAs present more than 200 nucleotides in length and also are involved in plant development and stress responses (Zhao et al., 2016). Lastly, circRNAs are a class of endogenous ncRNAs characterized by covalently closed structures without 5′ or 3′ ends (Bhogireddy et al., 2021). Rao et al. (2022b) investigated the tomato response to HS and they reported that plants improve their HS tolerance through Hsf-mediated transcriptional regulation of miR169s. HsfA1a, HsfA2 and HsfA7a have a key role in HS response and they also bind to the promoters of miR169, leading to transcriptional enhancement of miR169s. Enhanced accumulation of miR169s reduces the levels of the Sly-NF-YA9/A10 (Nuclear Factor-YA class of transcription factors) that leads to enhancement of the expression of HS-related genes like HsfA2, HsfA3 and HsfA7s. In a previous work, Rao et al. (2020) conducted a comprehensive analysis and they identified 18 miR169 precursors family members. Shi et al. (2019) investigated the role of the miR319d under HS, which was reported to acts as an essential regulator of gene expression during plant development and under stress conditions. Tomato plants showing its overexpression presented enhanced thermotolerance as consequence of an altered expression of several heat-related key genes (HsfA1a, HsfA1b and Hsp90) and genes involved in ROS signal transduction (ZAT12 and ZAT10).

Not only epigenetic modifications, but also post-transcriptional and post-translational regulations are reported to contribute to the molecular response to HS. Among the post-transcriptional regulations, high temperatures strongly affect splicing events of many genes (Kannan et al., 2018; Ling et al., 2021). Alternative splicing (AS) is a process in which two or more different transcripts are produced from one pre-mRNA molecule, thus affetting the availability and/or abundance of different kind of proteins. Different AS types can be classified, based on the action mechanisms: exon skipping, intron retention, alternative 5’ splice site selection and alternative 3’ splice site selection (Rosenkranz et al., 2022). Hu et al. (2020) studied the acclimatation to HS regulated by the AS of the HsfA2 in tomato, whose gene presents two introns. While the full or partial retention of the intron 1 occurs rarely, intron 2 is subjected to AS through full, partial or no splicing event. The complete and partial retention of the intron 2 comports the production of the HsfA2-Iα and HsfA2-Iγ splicing variants, and the abundance of the three isoforms depends on temperatures. Indeed, HsfA2-Iα was found to be mostly produced under severe heat stress, while HsfA2-Iγ and HsfA2-II under mild heat stress, thus evidencing its importance in the HS response. In addition, Keller et al. (2017) conducted a genome-wide study in tomato pollen, evidencing that more than 76% of these genes were subjected to AS based on intron retention or exon skipping under high temperatures compared with those of control conditions, thus identifying AS as a new HS regulatory layer for genes with a constitutive expression pattern. On the other hand, in the post-translational modifications of proteins, small label molecules including acetyl groups, phosphoric acids, lipids and small peptides are added to the target protein in response to a stress, thus inducing alterations of its location, stability or function. Five types of label molecules are reported in response to HS: ubiquitin, small ubiquitin-like modifiers (SUMOs), protein kinase, HATs and HDACs, the last two of which were already discussed. Ubiquitins are conjugated to a protein substrate by binding the lysine residues, and this mechanism is named ubiquitination. This labelling is mediated by three enzymes namely ubiquitin activating enzyme (E1), ubiquitin conjugating enzyme (E2) and ubiquitin ligase (E3). Among these, the E3 ligase genes are induced by HS. Zhang et al. (2021) studied the tomato carboxyl terminus of the HSC70-interacting proteins (CHIP), that is a conserved chaperone-dependent ubiquitin E3 ligase that targets misfolded proteins. SlCHIP was expressed under HS, and its silencing comported a reduction of the tomato basal thermotolerance, of photosynthetic activity and the accumulation of highly ubiquitinated insoluble protein aggregates. They found that the SlCHIP was involved in the HS response by targeting degradation of misfolded proteins that were generated under high temperatures. Another post-translational modification is the sumoylation performed by SUMOs, whose enzymatic mechanism is similar to that of ubiquitination (Guerra et al., 2015; Ghimire et al., 2020). Even in this case, labelling is mediated by three enzymes: SUMO activating enzyme 1 (SAE1 or E1), SUMO conjugating enzyme 1 (SCE1 or E2) and SUMO-protein ligase (E3). Zhang et al. (2018a) found that overexpression of SlSIZ1, a well-characterized SUMO E3 ligase, enhanced heat tolerance by regulating the activities of HsfA1 and increasing the content Hsp70. Indeed, under high temperatures, SlSIZ1 reduced the accumulation of ROS and induced the transcription of Hsfs and Hsps, among which Hsp70. In addition, it also interacts with SlHsfA1 to mediate the sumoylation of the master regulator thus enhancing tomato thermotolerance. Finally, protein phosphorylation mediated by protein kinase and phosphatase is a major post-translational modification, affecting protein function, localization, stability and interaction in response to heat stress (Han et al., 2022). Yu et al. (2019) investigated the role of a mitogen-activated protein kinase (MAPK3) under heat stress in tomato. They generated a knockout (KO) in SIMAPK3 mutant that was compared to wilt type tomato plants. Interestingly, slmapk3 KO mutants reduced the overproduction of ROS under heat stress thus evidencing higher thermotolerance than to the wilt type. By contrast, Ding et al. (2018) studied the role of the SlMPK1 and they found that it is a negative regulator of thermotolerance in tomato. Indeed, transgenic tomato plants presenting the SlMPK1 silenced gene enhanced the HS response, whereas its overexpression induced lower tolerance with a decrease of antioxidative enzyme activities and an increase of ROS. Lastly, Hu et al. (2021) investigated the role of the calcium-dependent protein kinases (CPKs) 28 in response to HS in tomato. After generating tomato cpk28 mutants using a CRISPR-Cas9 gene editing approach, they observed that the silencing of this gene comported an increase in ROS and protein oxidation and a decrease in the antioxidant enzymes activity, thus evidencing the positive function for CPK28 in the regulation of thermotolerance.

## 9 Future perspectives and open questions

**Figure 6:**
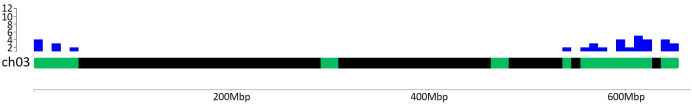
Distribution of Hsfs, Hsps, flower-, pollen- and fruit set-related genes mapping on chromosome 3. Green boxes represent regions where these genes map. Blue histograms indicate the number of genes mapping into a chromosome region of 1 Mbp. There are 30 genes in the distal end of the chromosome, such as one Hsf, 20 Hsps, FA and FUL2 genes, and nine pollen-related genes, among which SlNCED1. Graphical visualization was performed by using the ChromoMap R package (Anand and Rodriguez Lopez, 2022).

Plants usually face several biotic and abiotic stresses, which limits their performances in terms of both production and fruit quality, leading to negative ecological, economic and societal impacts. Among these, HS represents one of the main threats that adversely affects crops worldwide. A major future challenge for agriculture relies in the mitigation of climate change effects on crop production due to the rise in temperatures above the optimum, which leads to high yield losses. In this review, we decided to focus on the impact of HS on the reproductive stages of tomato, which represents one of the major horticultural crops in the world. Indeed, high temperatures mainly occur during these stages and dramatically affect organs like flowers, pollen and fruits that determine the final yield. Plants respond to stress at the molecular level by DNA sequences adaptive variations, transcriptional regulation, post-transcriptional and post-translational modifications of stress-related genes and proteins. In the literature, a high number of genes (Hsfs, Hsps, flower-, pollen- and fruit set-related) were reported to be involved in both reproduction mechanisms and HS response and we summarized and described them in the present review. From the position on tomato chromosomes of the 393 genes we focused on (listed in **Supplementary Table 4**), it is evident that there are hotspots of genes potentially affecting the response to HS (**Supplementary Figure 1**), as shown in **Figure 6** for chromosome 3. Interestingly, most hotspots regions identified co-localize with a high number of QTLs, such as those for stigma exertion, numbers of flowers, numbers of fruits (Graci et al., 2023).

Altogether, these findings might be represent a useful starting point for all the researchers interested in studying the response to HS in tomato, even though the plethora of genes here reported does not exhaustively describes it. As a whole, the variability of these genes could be further investigated by high-throughput phenotyping and genotyping platforms to discover functional mutations in coding and/or regulatory regions and identify new QTLs associated to yield-responsive plant traits that could determine contrasting tolerant/susceptible phenotypes. Indeed, the identification of genetic markers may improve the selection for key traits and their application in breeding programs. In addition, the available genome editing technologies, like CRISPR-Cas9, VIGS and other gene editing and silencing approaches, are valuable strategies to validate the function of candidate genes, thus allowing to understand plant molecular mechanisms in responses to HS. The study of overexpressed and silenced lines of poorly investigated and/or unknown genes through genetic transformation and their phenotyping in comparison with the wilt type would promote a more direct and accurate analysis of gene function. However, the functional study is not limited to the genes themselves as they usually interact in a complex network in which genes are both regulated by transcriptional factors and also encode downstream proteins, in a cascade of genes that could improve the response to HS. In addition, it should be also considered the functions and interactions of important epigenetic regulatory factors in the plant HS response. Whereas numerous studies focused on histone methylation and acetylation are reported, works on other epigenetic and post-translational modifications such as phosphorylation, ubiquitination, and SUMOylation are scarce and response mechanisms still remain unclear. Moreover, most methylation studies involved DNA while little is known about RNA methylation in response to HS. An integrative approach of these advanced technologies will permit to prioritize some of these genes and investigate their network, with the final aim of exploiting them in precise breeding approaches to obtain new plant materials able to successfully face climate changes.

## Author contributions

SG and AB conceived the review topic and outline. SG drafted the manuscript; AB revised the manuscript. All authors contributed to the article and approved the submitted version.
