# Supplementary material for: Tomato plant response to heat stress: a focus on candidate genes for yield-related traits
Source: Front Plant Sci. 2024 Jan 8;14:1245661. doi: 10.3389/fpls.2023.1245661 (PMC10800405; doi:10.3389/fpls.2023.1245661)
Supplement: Supplementary file 1 [file DataSheet_1.docx]

Supplementary Material

Tomato plant response to heat stress: a focus on candidate genes for yield-related traits

Salvatore Graci, Amalia Barone*

*** Correspondence:** Corresponding Author: ambarone@unina.it

# Supplementary Figure


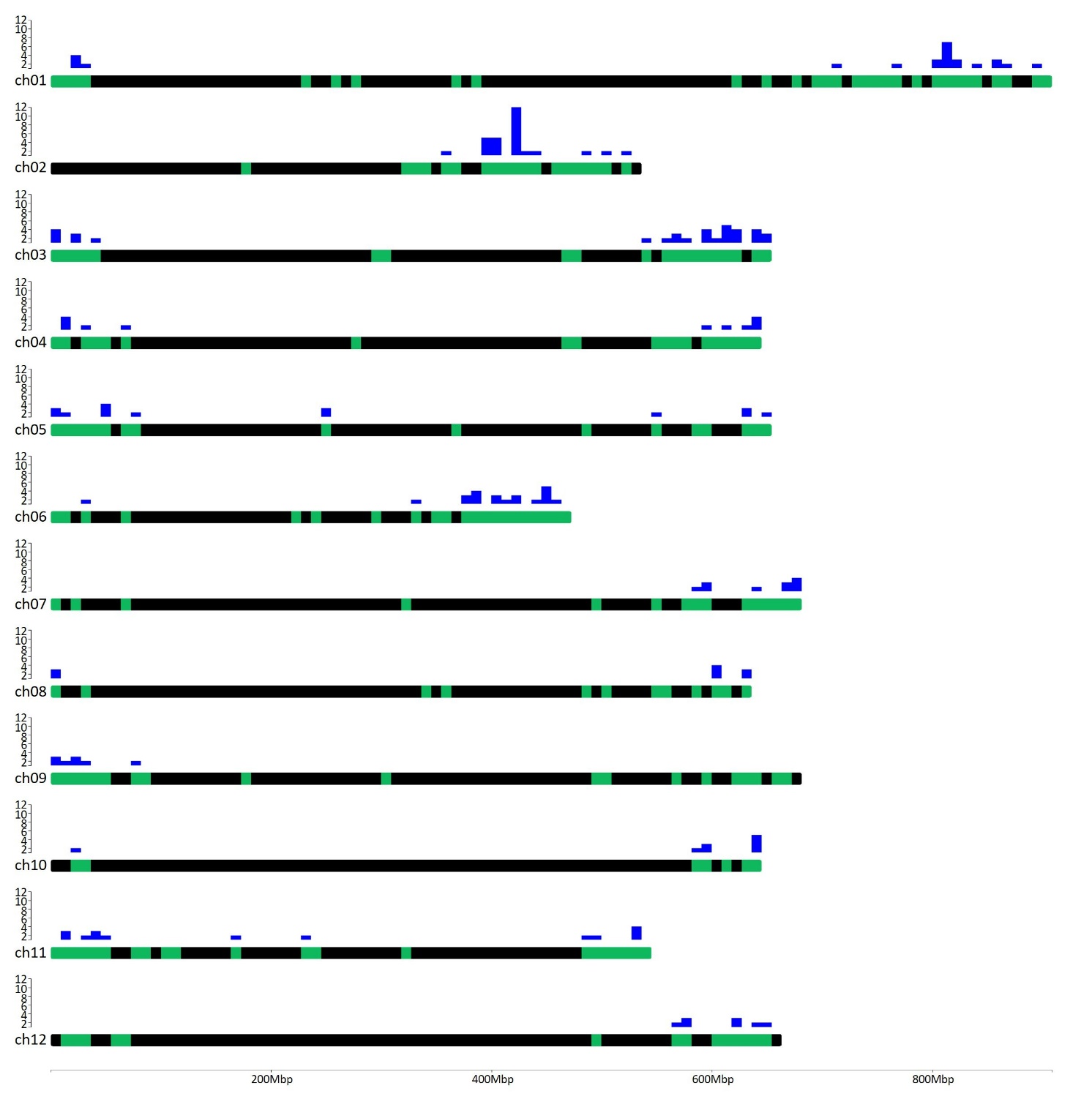


**Supplementary Figure 1** - Tomato chromosome distribution of Hsfs, Hsps, flower-, pollen- and fruit set-related genes. Green boxes represent regions where the reported genes are located. Blue histograms indicate the number of genes mapping into a chromosome region of 1 Mbp. Graphical visualization was performed by using the ChromoMap R package (Anand and Rodriguez Lopez, 2022).
